# Improving performance, reproduction, and immunity in laying Japanese quail with algal derivatives

**DOI:** 10.1016/j.psj.2023.103295

**Published:** 2023-11-17

**Authors:** Hassan Habibi, Enayat Rahmatnejad, Sayyed Sattar Tohidifar, Alireza Afshar, Ali Kameli, Maryam Jafari, Mehdi Mohammadi

**Affiliations:** ⁎ Department of Animal Science, Faculty of Agriculture and Natural Resources, Persian Gulf University, Bushehr 75169, Iran; † Department of Clinical Sciences, Faculty of Veterinary Medicine, University of Shahrekord 8818634141, Shahrekord, Iran; ‡ The Persian Gulf Marine Biotechnology Research Center, The Persian Gulf Biomedical Sciences Research Institute, Bushehr University of Medical Sciences, Bushehr, Iran; § Graduated Master of Science in Medical Mycology, Kerman University of Medicine Science, Kerman, Iran; # Department of Marine Biotechnology and Environment, Persian Gulf Research and Studies Center, Persian Gulf University, Bushehr, Iran

**Keywords:** algal derivatives, immune response, laying Japanese quail, reproduction

## Abstract

We investigated the effect of the Persian Gulf algae derivatives, namely phycocyanin (**PC**) and fucoidan (**FUC**), on the performance, reproductive traits, and immune responses of laying Japanese quails. A completely randomized design was used to distribute 250 six-wk-old Japanese quails with an average body weight of 215 ± 10 g into 5 treatments, 5 replicates, and 10 birds in each replicate over a 5-wk period. Unlike the control groups, the treatment groups received drinking water supplemented with PC and FUC at concentrations of 20 or 40 mg/L, denoted as PC20, PC40, FUC20, and FUC40, respectively, while all birds were provided with identical feed. Supplemental algal derivatives notably improved hen day egg production (**HDEP**), egg mass, and feed conversion ratio (**FCR**) compared to the control group (*P* < 0.01). Incorporating PC and FUC had no significant effect on the weight of males' testes or the weight and length of hens' oviducts. Additionally, the experimental treatments had no impact on the chicks' hatching weight. The supplementation of PC and FUC resulted in increased fertility (*P* = 0.038) and hatchability (*P* < 0.001) rates, with the exception of fertility in the PC40 group. The effect of the experimental treatments on immune responses was largely not statistically significant, except in the case of ND. Specifically, the experimental treatments resulted in increased (*P* = 0.033) antibody titers against ND when compared to the control group, with the exception of FUC20. Supplemental algal derivatives significantly (*P* < 0.01) reduced total cholesterol, creatinine, and triglycerides (except in the case of PC20). Overall, these findings underscore the potential of algal derivatives to enhance quail performance, reproductive traits, and immune responses.

## INTRODUCTION

Poultry products serve as a significant means of providing economical animal protein across the globe. However, with the growing human population, there's a need to improve poultry production for a steady supply of protein (Nhlane et al., 2021). This necessitates the development of strategies that can potentially enhance the productive and reproductive performance of poultry. Nutritional approaches encompass the use of diverse feed additives such as exogenous enzymes (Whiting et al., 2017; Anderson et al., 2023), probiotics (Saeeda et al., 2023), prebiotics (Froebel et al., 2019; Rehman et al., 2020), and phytogenic supplements (Hafeez et al., 2016; Cimrin et al., 2020; Habibi et al., 2022, 2023), all of which have been examined to enhance poultry production.

Phytogenic feed additives can be introduced into animal feed or administered through water as extracts. Algae and their derivatives, when employed as phytogenic feed additives, are notable for their bioactive properties (Zheng et al., 2020). They contain an abundance of phenolic compounds, flavonoids, phycocyanin (**PC**), fucoidan (**FUC**), and various other bioactive substances (Farag et al., 2016; Zheng et al., 2020). These compounds have been well-documented for their diverse biological activities, including antioxidative, anti-inflammatory, antimicrobial properties, prebiotic properties (O'Doherty et al., 2010; Lordan et al., 2013; Méresse et al., 2020).

A noteworthy group of biomolecules is phycobiliproteins, which are known for their antioxidant properties and are found in cyanobacteria and various algae, including Rhodophytes, Cryptomonads, and Glaucocystophytes (Grover et al., 2021; Lauceri et al., 2023). Arthrospira platensis, commonly known as *Spirulina*, is a globally cultivated cyanobacteria that contains phycocyanin, a member of the phycobiliprotein family. Phycocyanin has shown promise in enhancing the performance, antioxidant levels, reducing inflammatory responses, and controlling intestinal pathogens, thereby contributing to the overall health improvement of growing rabbits during the summer season (Abdelnour et al., 2020). Fucoidans, sulfated and rich in fucose, are another bioactive polymers found in brown macroalgae and can be easily extracted from their cell walls (Lauceri et al., 2023). Indeed, previous research has showcased the capacity of FUC and similar compounds to mitigate liver injury by suppressing the inflammatory pathway in mice (Li and Ye, 2015) and to enhance overall health through their prebiotic properties (O'Doherty et al., 2010; Yoo et al., 2019).

It has been shown that dietary supplementation with marine-derived polysaccharides can enhance broilers' immunity (Zhao et al., 2021) and improve productive performance, egg quality, and antioxidant capacity in late-phase laying hen (Guo et al., 2020). The later effect was evidenced by increased activity of serum total superoxide dismutase, liver, and jejunal catalase, along with a decrease in jejunal malonedialdehyde concentration (Guo et al., 2020). Moreover, the previous study demonstrated the positive effect of algae and their derivatives on reproductive performance of laying poultry. For instance, the fertility and hatchability percentages of eggs produced by local laying hens fed diets containing *Spirulina* were significantly higher compared to those of the control group (Mariey et al., 2012).

Despite their diverse biological activities, there is a notable absence of studies regarding the utilization of PC and FUC in laying Japanese quail. Consequently, this research was undertaken to address this knowledge gap and assess whether these algal derivatives can influence the productive performance, reproductive traits, blood profiles, and immune responses of laying Japanese quail.

## MATERIALS AND METHODS

The experiment was carried at the experimental facility of Persian Gulf University, Bushehr, Iran. All protocols employed in this research received approval from the Local Experimental Animal Care Committee within the Department of Animal Science, Faculty of Agriculture and Natural Resources, Persian Gulf University, Bushehr, Iran (contract number 98/1001).

### Birds, Treatments, and Management

In total, 250 Japanese quails with an average body weight of 215 ± 10 g at 6 wk of age in the laying phase were randomly divided into 5 treatment groups in a complete randomized design experiment with 5 treatments having 5 replicates of 10 birds (eight hens and 2 males) each.

The experiment lasted 5 wk. The PC (from *Spirulina platensis*) and FUC (from brown seaweed) sourced from the coastlines of the Persian Gulf were purchased from Zarband Co. (Yasuj, Iran). The study involved the addition of 2 different concentrations of PC and FUC extracts to the drinking water, resulting in the following 4 treatment groups, which were compared to a control group receiving water with no additives: 20 mg/L phycocyanin (PC20), 40 mg/L phycocyanin (PC40), 20 mg/L fucoidan (FUC20), and 40 mg/L fucoidan (FUC40). The composition of the basal diet ingredients was formulated to meet requirements by the NRC (1994), and the details are presented in Table 1. At every stage of the trial, the birds had unlimited access to feed and drink. The lighting regimen followed a schedule of 16 h of light and 8 h of darkness, and the temperature inside the housing facility was maintained at a constant 20°C, whereas, the relative humidity was maintained at approximately 60 to 70%. All quails were raised in wire batteries under uniform management, hygiene, and environmental conditions.

**Table 1:** Composition and calculated analysis of the basal diet (6–11 wk).

Item	Content (g/kg)
Ingredient	
Yellow corn	530.0
Soybean meal (44%)	356.3
Vegetable oil	35.0
Dicalcium phosphate	11.0
Limestone	58.0
Sodium chloride	3.5
dl-Methionine	1.2
Vitamin-mineral premix^1^	5.0
Total	1000.0
Calculated analysis (%)	
Metabolizable energy (kcal/kg)	2900
Crude protein	19.97
Calcium	2.50
Available phosphorous	0.36
Sodium	0.15
Lysine	1.10
Methionine	0.44
Methionine + Cystine	0.77
Threonine	0.77

1 Vitamin-mineral premix: supplied the following per kilogram of diet: vitamin A, 10,000 IU; vitamin D_3_, 3,000 IU; vitamin E, 20 IU; vitamin K_3_, 2 mg; thiamin, 2 mg; riboflavin, 8 mg; niacin, 50 mg; calcium d-pantothenate, 20 mg; pyridoxine, 4 mg; folic acid, 1 mg; cyanocobalamine, 0.06 mg; biotin, 0.2 mg; choline chloride, 320 mg; Fe (FeSO_4_·7H_2_O), 60 mg; Mn (MnSO_4_·H_2_O), 80 mg; Zn (ZnO), 60 mg; Cu (CuSO_4_·5H_2_O), 10 mg; Se (Na_2_SeO_3_), 0.2 mg; I (KIO_3_), 0.3 mg.

### Growth Performance

The quails were individually weighed at the beginning and end of the experiment and monitored daily for signs of illness or death. Performance measures, including hen day egg production (**HDEP**), egg weight, egg mass, daily feed intake (**FI**), and feed conversion ratio (**FCR**), were assessed. Daily feed intake was recorded in grams of feed consumed per bird per day, and mortality rates were carefully documented. Feed conversion ratio (g feed/g egg) was determined according to the egg mass value divided by the quantity of feed consumption. Eggs were collected daily and HDEP was calculated using the formula by North and Bell (1990), which is expressed as a percentage and defined as (number of eggs produced on each day/number of hens alive on that day) × 100. Egg count and egg weight were recorded on a daily basis, and egg mass (egg number × egg weight) was subsequently calculated.

### Reproductive Performance and Sexual Organs Development

At the end of the experiment, in order to assess certain reproductive traits, 1 female and 1 male bird were selected from each pen and humanely euthanized through cervical dislocation. After slaughtering, the abdominal cavity of each bird was opened, and the weight of the oviducts in hens and the testes in males were determined as a percentage of their live body weights. Additionally, the length of the hen's oviduct was measured in centimeters. Also, a total of 15 eggs from each treatment group were collected at the last week of the experimental period. These eggs were then subjected to a disinfection process and placed in an incubator. During the initial 14 d of incubation, the incubated eggs were maintained at a temperature of 37.5°C and a relative humidity of 65%. Subsequently, on the 15th day of incubation, the eggs were transferred to a hatchery machine and maintained at a temperature of 37.4°C and a relative humidity of 70% until hatching. After hatching, the number of chicks that successfully hatched was counted, and any eggs that did not hatch were also counted. This information was used to calculate both fertility and hatchability rates. Fertility was calculated as the sum of the number of hatched chicks and the number of fertile, nonhatched eggs, divided by the total number of eggs initially set in the incubator, and then multiplied by 100 to express it as a percentage. Hatchability, on the other hand, was determined based on the chicks that hatched from the fertile eggs. Moreover, the initial weight of hatched chicks was also recorded.

### Blood Profile and Immune Responses

At the end of the experiment, blood samples were collected from 5 hens and 5 males in each treatment group by severing their jugular veins. Each bird provided 2 blood samples; the first sample was collected in a nonheparin tube, and the blood serum was separated by centrifugation at 3,000 rpm for 15 min and stored at −20°C for measuring humoral immune responses, while the second sample was obtained in a heparinized tube and stored at +4°C for subsequent hematological parameter examination. Complete blood count (hemoglobin, WBC, RBC, PCV, and platelets) assessment was performed on Sysmex hematology analyzer (Model XP100; Sysmex Suisse AG, Yverdon-les-Bains, Switzerland). Lymphocyte, monocyte, eosinophil, and basophil were counted manually under the microscope.

To investigate humoral immune responses, all groups were vaccinated with Newcastle (**ND**) and avian influenza (**AI**) killed vaccine (subtype H9N2) subcutaneously in the breast at 10 d of age. Humoral immune responses were investigated by detecting serum antibody levels against ND and AI by hemagglutination inhibition test as described by Habibi et al. (2022).

Triglyceride, total cholesterol, high-density lipoprotein cholesterol (**HDL**), low-density lipoprotein cholesterol (**LDL**), total protein, albumin, uric acid, creatinine, calcium, phosphorous, alanine aminotransferase (**ALT**), aspartate aminotransferase (**AST**), and alkaline phosphatase (**ALP**), activities were assessed using specific kits (Pars Azmoun, Tehran, Iran) and a spectrophotometer (BT1500).

### Statistical Analysis

Data obtained from the experiment were analyzed according to a completely randomized design using the GLM procedure of SAS Institute Inc. (SAS, 2010). Duncan's multiple comparison test was used to compare the treatment means at the probability level of 5% (*P* < 0.05). The results are provided as mean values with their respective standard errors.

## RESULTS

### Performance

**Figure 1:**
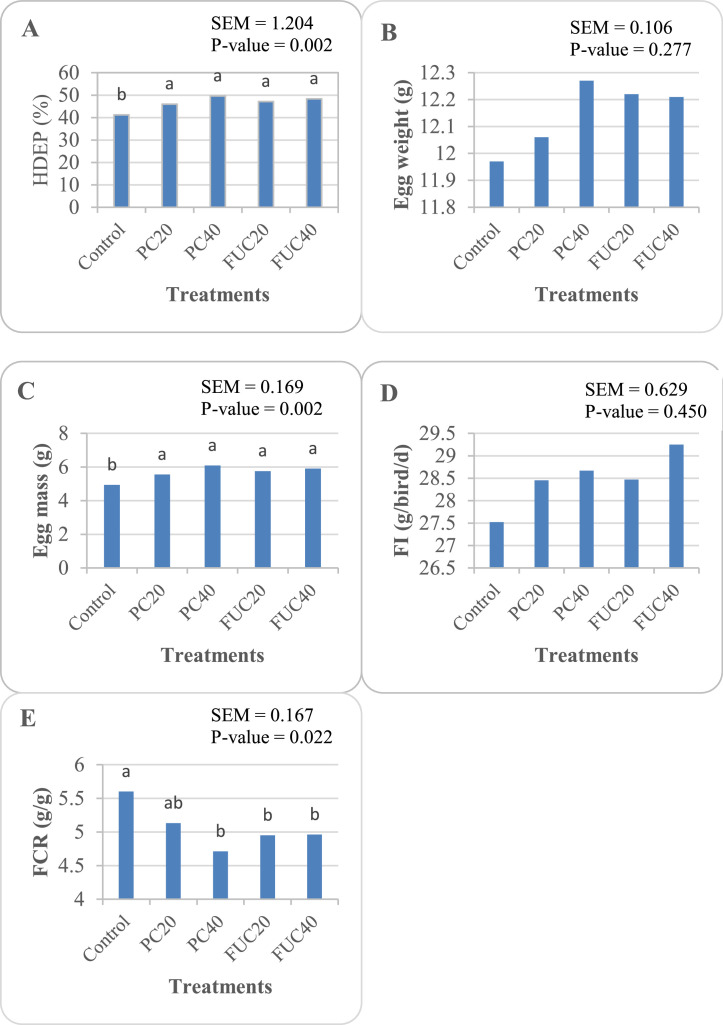
Effect of different treatments on productive performance of laying Japanese quails. HDEP, hen day egg production; FI, daily feed intake; FCR, feed conversion ratio; PC20, phycocyanin 20 mg/L; PC40, phycocyanin 40 mg/L; FUC20, fucoidan 20 mg/L; FUC40, fucoidan 40 mg/L. ^a,b^Means with no superscript letters or a common superscript letter are not significantly different (*P* > 0.05).

Figure 1 displays the effect of various treatments on the performance of laying Japanese quails throughout the 5-wk duration. Significant improvements (*P* < 0.01) were noted in HDEP, egg mass, and FCR (with the exception of PC20) when compared to the control group (Figure 1A, C, E). The highest HDEP and egg mass, along with the lowest FCR, were observed in the PC40 treatment. However, it is worth noting that supplementing PC and FUC did not have a significant effect on FI and egg weight (*P* > 0.05).

### Reproductive Performance and Sexual Organs Development

**Figure 2:**
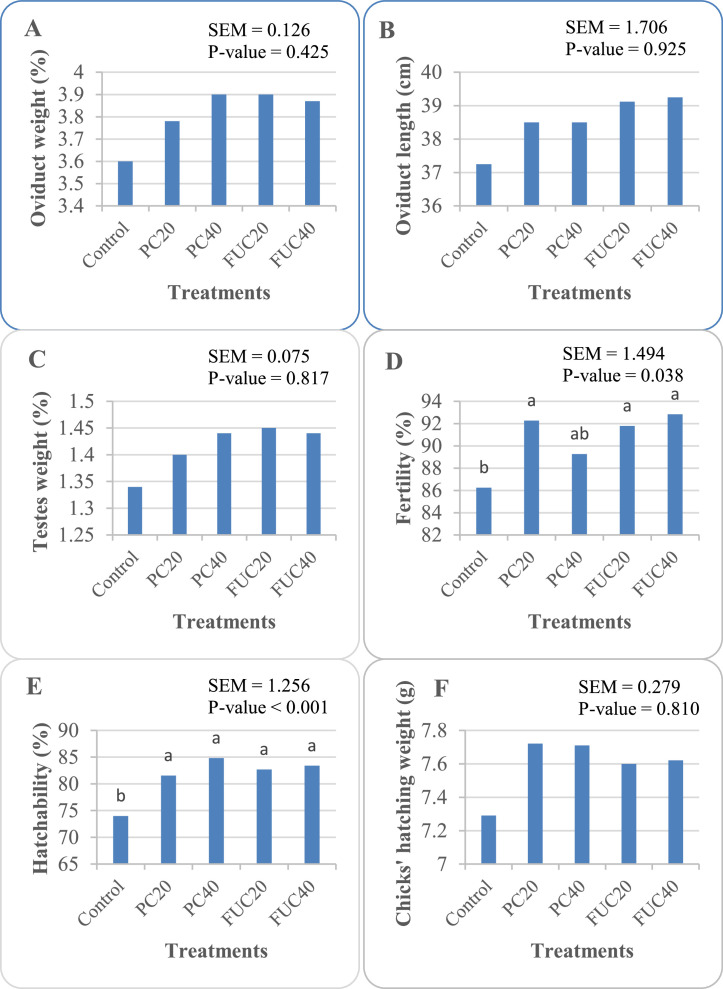
Effect of different treatments on reproductive performance and sexual organs development of laying Japanese quails. PC20, phycocyanin 20 mg/L; PC40, phycocyanin 40 mg/L; FUC20, fucoidan 20 mg/L; FUC40, fucoidan 40 mg/L. ^a,b^Means with no superscript letters or a common superscript letter are not significantly different (*P* > 0.05).

The reproductive performance and sexual organs development at the end of experiment are shown in Figure 2. The study's results suggest that while the inclusion of PC and FUC appeared to stimulate the development of sexual organs (Figure 2A–C), it did not lead to any significant changes in the weight of males' testes, or the weight and length of hens' oviducts (*P* > 0.05). Furthermore, the chicks' hatching weight (Figure 2F) remained unaffected by the experimental treatments (*P* > 0.05). As indicated in Figure 2D, E, the supplementation of additives resulted in increased fertility (*P* = 0.038) and hatchability (*P* < 0.001) rates, with the exception of fertility in the PC40 group.

### Blood Profile and Immune Responses

As indicated in Table 2, the influence of the experimental treatments on immune responses was largely not statistically significant, except in the case of ND. Specifically, the experimental treatments resulted in increased antibody titers against ND when compared to the control group (*P* = 0.033), with the exception of FUC20, which did not show a significant increase (*P* > 0.05). Additionally, the experimental treatments had no significant impact on antibody titers against AI (*P* > 0.05).

**Table 2:** Effect of different treatments on immunity responses of laying Japanese quails^1^.

Item	Control	PC20	PC40	FUC20	FUC40	SEM	*P* value
Hb (g/dL)	10.72	11.04	11.02	10.98	11.27	0.033	0.837
RBC (×10^6^/µL)	2.73	3.01	2.92	2.92	2.83	0.156	0.764
WBC (×10^3^/µL)	17.15	17.24	17.87	17.57	18.21	0.900	0.909
Platelets (×10^3^/µL)	34.80	34.32	35.02	35.40	35.20	1.116	0.966
PCV (%)	33.55	337.72	33.22	33.25	33.45	0.996	0.995
Eosinophil (%)	0.29	0.30	0.28	0.30	0.34	0.035	0.784
Basophil (%)	0.23	0.25	0.27	0.27	0.26	0.038	0.951
Lymphocyte (%)	60.50	61.75	62.25	62.00	63.25	0.905	0.353
Monocyte (%)	1.40	1.32	1.35	1.40	1.37	0.106	0.983
ND (log2)	5.00^b^	6.50^a^	6.50^a^	5.75^ab^	6.5 0^a^	0.359	0.033
AI (log2)	5.00	6.25	6.00	6.00	6.75	0.397	0.081

1 PC20, phycocyanin 20 mg/L; PC40, phycocyanin 40 mg/L; FUC20, fucoidan 20 mg/L; FUC40, fucoidan 40 mg/L; Hb, hemoglobin concentration; RBC, red blood cell; WBC, white blood cell; platelets; PCV, packed cell volume. The eosinophil, basophil, lymphocyte, and monocyte units are expressed as a percentage of the total white blood cell count. The ND and AI refer to the antibody titer against Newcastle disease and avian Influenza viruses, respectively.

a,b Means in the same row with no superscript letters or a common superscript letter after them are not significantly different (*P* > 0.05). SEM, standard error of the mean.

The effect of the treatments on serum profile is shown in Table 3. The investigated treatments were found to significantly decrease total cholesterol (*P* = 0.001) and creatinine (*P* = 0.007). Algal derivatives also considerably decreased triglycerides (*P* = 0.003), with the exception of PC20, which decreased but not significantly (*P* > 0.05) compared to the control. Other serum indices like HDL, LDL, total protein, albumin, uric acid, calcium, phosphorous, AST, ALT, and ALP were not significantly (*P* > 0.05) affected by the treatments.

**Table 3:** Effect of different treatments on serum biochemistry profile of laying Japanese quails^1^.

Item	Control	PC20	PC40	FUC20	FUC40	SEM	*P* value
Triglyceride (mg/dL)	233.25^a^	200.66^ab^	187.00^bc^	167.00^bc^	157.50^c^	11.96	0.003
TC (mg/dL)	210.50^a^	177.00^b^	167.00^b^	170.25^b^	150.25^b^	7.80	0.001
HDL (mg/dL)	39.50	46.33	49.75	54.25	56.00	4.04	0.067
LDL (mg/dL)	106.00	121.66	127.50	116.78	122.00	6.21	0.200
Total protein (g/dL)	3.71	4.05	4.01	4.12	4.26	0.20	0.463
Albumin (g/dL)	2.46	2.85	3.11	2.62	2.94	0.15	0.068
Uric acid (mg/dL)	2.75	2.40	2.30	2.34	2.30	0.16	0.305
Creatinine (mg/dL)	0.49^a^	0.36^b^	0.33^b^	0.37^b^	0.35^b^	0.02	0.007
Calcium (mg/dL)	13.76	14.38	12.27	13.58	13.74	0.60	0.301
Phosphorous (mg/dL)	7.14	7.48	7.77	8.25	8.22	0.54	0.560
AST (U/L)	33.73	34.25	32.65	32.87	33.95	1.98	0.971
ALT (U/L)	20.52	20.45	20.60	20.45	20.52	0.44	0.999
ALP (U/L)	121.90	123.75	120.30	121.97	119.47	1.86	0.547

1 PC20, phycocyanin 20 mg/L; PC40, phycocyanin 40 mg/L; FUC20, fucoidan 20 mg/L; FUC40, fucoidan 40 mg/L; Hb, hemoglobin concentration; RBC, red blood cell; WBC, white blood cell; platelets; PCV, packed cell volume. The eosinophil, basophil, lymphocyte, and monocyte units are expressed as a percentage of the total white blood cell count. The ND and AI refer to the antibody titer against Newcastle disease and avian Influenza viruses, respectively.

a–c Means in the same row with no superscript letters or a common superscript letter after them are not significantly different (*P* > 0.05). SEM, standard error of the mean.

## DISCUSSION

In this experiment, we evaluated the impact of PC and FUC on the performance, reproductive characteristics, and immune responses of laying Japanese quails. Our results unveiled noteworthy effects of these extracts on multiple parameters, underscoring their promise as beneficial dietary supplements for laying quails.

Various levels of PC and FUC demonstrated enhancements in HDEP, egg mass, and FCR, with the exception of PC20, as depicted in Figure 1. Hence, these extracts could potentially serve as viable candidates for enhancing quail performance. Limited research has been conducted on the effects of PC and FUC in poultry nutrition, particularly in the context of laying quails. Nonetheless, in alignment with these findings, a study by Mariey et al. (2012) reported that the dietary inclusion of *Spirulina platensis* (at 0.10, 0.15, or 0.20%) led to improvements in egg production, egg weight, and egg mass in local layer hens in Egypt. Furthermore, Draper et al. (2016) observed that dietary FUC supplementation led to increased FI and decreased FCR in weaning pigs, while Yang et al. (2022) found that adding 0.1, 0.3, and 0.5% FUC to the basal diet significantly lowered FCR in goat kids, and Park et al. (2018) reported a positive linear correlation between increased dietary supplementation of *Spirulina platensis* and improved FCR in broiler chickens, both during d 8 to 21 and overall. Also, Yang et al. (2022) found that dietary FUC supplementation did not yield significant effects on FI during the first 14 d in castrated male kids. Results showed that feed intake, egg production, egg weight and mass, and feed conversion ratio were not significantly affected by the dietary treatments. Also, as per the findings reported by Curabay et al. (2021) and Tufarelli et al. (2021), the incorporation of 1 and 2% SP into the diets of laying hens did not result in any significant alterations in performance parameters, which is inconsistent with the current study. Consistent with our research findings, Abdel-Warith et al. (2021) had previously reported that the final weight, weight gain, specific growth rate, and survival rate of Nile tilapia, exposed to toxic atrazine, increased significantly when provided with a diet containing FUC. Furthermore, a positive effect on the performance of broiler chickens has been reported with the use of *Spirulina* PC, as indicated by Omar et al. (2022). Nevertheless, in contrast to our own research outcomes, Abouelezz (2017) incorporated *Spirulina platensis* into the feed (1%) and drinking water (0.25%) of laying Japanese quails, finding no significant difference in the birds' egg production. Similarly, Hajati et al. (2020) did not observe any impact on the laying performance of Japanese quails when they tested various dietary levels of *Spirulina platensis*.

Fucoidan and similar compounds, such as linamarin, found in brown seaweeds are known to resist hydrolysis in the upper digestive tract, leading to their consideration as a prebiotic form (Corino et al., 2021; Zhu et al., 2021). Consequently, it can be inferred that there may be a connection between the prebiotic properties of FUC and the observed performance improvements in this study. The favorable outcomes observed with fucoidan in the current experiment may be attributed to immune responses (Jayawardena et al., 2022). However, as shown in Table 2, it is important to highlight that in our experiment, there was a lack of significant increases observed in various immune responses, including monocytes. Additionally, these positive effects may also be linked to the reduction of harmful bacteria such as *E. coli* in the digestive tract, as suggested by O'Doherty et al. (2010). The favorable impacts of phycocyanin on performance may be attributed to its multifaceted properties, including antioxidant, radical scavenging, anti-inflammatory, antiarthritic, hepatoprotective, antitumor, and immune-enhancing qualities (Liu et al., 2016; Chen et al., 2022). It is worth noting that phycocyanin is also a safe, natural antimicrobial product (Demirel et al., 2009; El-Araby et al., 2022). Furthermore, the positive impact of PC on performance may be attributed to the enhancement of birds' health, as indicated by the improved morphology of the small intestine, including greater villi length, increased goblet cell numbers, and an overall enhancement in absorption surface area. These factors likely contribute to improved nutrient digestibility and absorption (Omar et al., 2022). Research on PC in poultry nutrition is indeed limited, but it is noteworthy that phycocyanin is derived from *Spirulina*, and there have been studies conducted on *Spirulina*'s properties. Therefore, the improved performance observed in this study could potentially be attributed to the beneficial characteristics of *Spirulina*. Possible factors contributing to the inconsistency observed in the outcomes encompass the animals examined, the quantity and source of the algal derivatives employed, and the experimental circumstances.

Research regarding the effect of PC and FUC on poultry reproductive performance is indeed limited. However, in agreement with ours, the results of El-Ratel et al. (2023) indicated that *Spirulina* supplementation enhanced reproductive traits in rabbits. While the impact of phycocyanin on the development of sexual organs, such as oviduct weight and length, and testes weight, did not reach statistical significance, there was a numerical increase observed. The notable enhancements in hatchability are consistent with the findings of Mariey et al. (2012), who observed increased hatchability in laying hens fed a diet with a low level of *Spirulina* inclusion. Additionally, consistent with the results of our present study, it was discovered that the inclusion of 0.8 g of *Spirulina* per kilogram of diet improved hatchability in Sinai laying hens (Ismail et al., 2023). It is possible that the well-known antioxidant properties of algae derivatives explain the observed increases in hatchability, by improving oxidative state of embryos incubation, and fertility in Japanese quails (Hajati et al., 2021).

The administration of PC and FUC in this experiment led to enhancements in the immune responses of quails, even though statistically significant differences were not observed in all cases, with the exception being the antibody titer against ND. Our results regarding antibody titer against ND, are in accordance with the results of the study of Habibi et al. (2022), reporting antiviral properties of medicinal herbs.

As observed in studies conducted by Abdel-Warith et al. (2021) on the use of FUC in tilapia diet, Dogan et al. (2016) regarding the inclusion of Spirulina platensis in the diet of Japanese quail, and Omar et al. (2022) concerning PC in broiler chickens' diet, our current research highlights the promising potential of algae derivatives in the modulation of blood metabolites in quails. This is exemplified by the observed decrease in total cholesterol and triglyceride levels. Creatinine, a metabolic waste generated during protein metabolism in muscles, serves as an indicator of kidney function as it is excreted by the kidneys (Finn, 2016). In this study, we observed a significant decrease in serum creatinine levels in treated quails compared to the control group, suggesting efficient nephron function. The properties of PC and FUC, which enable them to capture free radicals, likely contribute to kidney protection, preventing damage to tubular and glomerular cells. This suggests that such derivatives may have beneficial effects on the health of quail. In contrast to our findings, it is important to mention that in an experiment conducted by (Omar et al., 2022), supplementation with different levels of PC in broiler chickens resulted in a linear increase in total proteins and albumin levels. Furthermore, in contrast to our findings, it is worth noting that in a study involving the inclusion of FUC in the diet of tilapia, a decrease in AST and ALT levels induced by toxic atrazine has been observed (Abdel-Warith et al., 2021). The discrepancies observed in the results may be attributed to several factors, including variations in the species of animals studied, the dosage and source of the algal derivatives used, and differences in experimental conditions.

## CONCLUSIONS

In conclusion, the quails that received algal derivatives in their drinking water exhibited noteworthy enhancements in HDEP, egg mass, and FCR when compared to the control group, with the PC40 treatment demonstrating the most favorable outcomes. Fertility and hatchability rates also increased with the supplementation of additives, except for PC40 regarding fertility. Furthermore, the experimental treatments demonstrated their efficacy in enhancing the birds' immune responses, as evidenced by increased antibody titers against ND, except for the FUC20 treatment, which did not show a significant increase. Furthermore, supplementation of PC and FUC led to reductions in serum cholesterol, creatinine, and triglyceride (except for PC20) levels. Overall, these findings underscore the potential of algal derivatives to enhance quail performance, reproductive traits, and immune responses.
